# Effects of COVID-19 contagion in cohabitants and family members on mental health and academic self-efficacy among university students in Sweden: a prospective longitudinal study

**DOI:** 10.1136/bmjopen-2023-077396

**Published:** 2024-03-12

**Authors:** Claes Andersson, Anne H Berman, Petra Lindfors, Marcus Bendtsen

**Affiliations:** 1 Department of Criminology, Malmo University, Malmo, Sweden; 2 Department of Psychology, Uppsala University, Uppsala, Sweden; 3 Department of Psychology, Stockholm University, Stockholm, Sweden; 4 Department of Health, Medicine and Caring Sciences, Linköping University, Linkoping, Sweden

**Keywords:** COVID-19, mental health, public health

## Abstract

**Objective:**

This study used causal inference to estimate the longitudinal effects of contagion in cohabitants and family members on university students’ mental health and academic self-efficacy during the COVID-19 pandemic.

**Design:**

A prospective longitudinal study including a baseline online measurement in May 2020, and online follow-ups after 5 months and 10 months. Participants were recruited through open-access online advertising.

**Setting:**

Public universities and university colleges in Sweden.

**Participants:**

The analytical sample included 2796 students.

**Outcome measures:**

Contagion in cohabitants and in family members was assessed at baseline and at the 5-month follow-up. Mental health and academic self-efficacy were assessed at the 5-month and 10-month follow-ups.

**Results:**

Mild symptoms reported in cohabitants at baseline resulted in negative mental health effects at follow-up 5 months later, and mild baseline symptoms in family members resulted in negative effects on academic self-efficacy at follow-ups both 5 and 10 months later.

**Conclusions:**

Notwithstanding the lack of precision in estimated effects, the findings emphasise the importance of social relationships and the challenges of providing students with sufficient support in times of crisis.

**STRENGTHS AND LIMITATIONS OF THIS STUDY**

- A prospective longitudinal study including a large sample of university students.
- Use of a causal model allowing examination of the effects of observational data.
- The estimates presented in the analysis should only be understood to represent effects if the causal model is appropriate.
- Low rates of severe symptoms, or deaths, in cohabitants and family members limit the possibility to fully explore the effects of negative consequences other than self-reported mild symptoms.
- Single items tend to align with the overall scores obtained from multi-item measures but are generally considered to have lower content validity, sensitivity and reliability.

## Introduction

Strong theoretical and empirical evidence support the causal impact of social relationships on mental health.^1^ A recent review shows that social relationships have a protective effect on health while lacking such relationships is linked to risk.^2^ Importantly, mental health is influenced by biopsychosocial processes, from a biological microlevel to a social macrolevel, with major pathways including social support, social influence, engagement and attachment, and access to resources and material goods.^3^ As for the importance of social relationships and mental health, illness or loss due to death, for instance, in cohabitants or family members, has consistently been found to have negative spillover effects on the mental health of individuals within the same social system.^4^ Moreover, social relationships and the health of cohabitants and family members are important for university students, a group that has been identified as vulnerable.^5^ For instance, students with lower-quality social support are more likely to experience mental health problems.^6^ Also, both positive mental health and academic self-efficacy have been associated with access to support from both friends and families.^7^

The global COVID-19 pandemic that started in early 2020 resulted in illness and increased death rates across the world.^8^ The multitude of stressors caused by the pandemic has been described as a trauma.^9^ Importantly, access to social relationships was restricted as behavioural measures to control the spread of the virus were introduced.^10^ These restrictions altered exchanges in relation to social networks, social support, social interaction and intimacy.^11^ In several countries, restrictions also implied lockdown of university campuses.^12^ An early review on the initial phase of the pandemic indicates an overall negative effect on students’ mental health.^13^ Cross-sectional findings suggest that knowing someone who has been infected had a negative impact on mental health in students in the USA.^14^ Moreover, contagion among family members negatively affected the mental health and ability to carry out studies among students in Spain.^15^ In contrast, a cross-sectional study from Sweden found no association between contagion in cohabitants and family members and mental health and academic self-efficacy, respectively, although students did report increased self-contagion.^16^ A possible explanation for the findings from Sweden may involve low frequency of contagion, severe illness and death among cohabitants and family members during the initial phase of the pandemic. Another explanation may relate to the fact that most students in Sweden live on their own or in their own rooms in shared accommodation.^16^ However, other factors explaining national differences may for instance involve the timing of a study, the level of contagion at these specific time points^17^ and cultural differences related to the high level of values related to rationality and self-expression.^18^ In addition, it is important to note that in Sweden measures to control the spread of the virus were based solely on voluntary measures and citizen responsibility. In contrast to a lock-down approach, this made it possible to maintain important social relationships and contacts if this was considered necessary.^19^ The Swedish Government has reported that the overall socioeconomic effects of the pandemic were less prominent in Sweden as compared with many other European countries.^20^

Considering the significance of social relationships in conjunction with the large proportion of individuals affected by COVID-19, there is a scarcity of studies, particularly longitudinal, about whether contagion via social relationships affected students’ mental health and academic self-efficacy. In relation to future pandemics and similar broad-ranging crises, it is valuable to identify the long-term effects of factors that may influence mental health and academic self-efficacy in university students, so that university management strategies and student healthcare can be adapted accordingly.

The overall objective of the current study is to investigate the longitudinal effects of contagion in cohabitants and family members on mental health and academic self-efficacy in university students. Specifically, the first aim was to estimate the effect of baseline contagion in cohabitants and family members on mental health and academic self-efficacy 5 and 10 months postbaseline. The second aim was to estimate the effects of contagion in cohabitants and family members at 5 months postbaseline, on mental health and academic self-efficacy 10 months postbaseline. Contagion in family members was expected to have effects on student mental health and academic self-efficacy at both follow-ups.

## Methods

### Setting and procedures

This study is based on an ongoing collaboration that involves the majority of the public higher education institutions (HEIs) in Sweden and is the Sweden-based partner in the WHO World Mental Health International College Student Initiative. Thus, the present longitudinal study was conducted in Sweden and includes one baseline online survey and two follow-ups. In May 2020, after vetting by the Swedish Ethical Review Authority (Ref. No. 2020-02109), a baseline cross-sectional survey was administrated through advertisements on the websites of ten public HEIs as well as the National Association of Student Unions. Interested students followed a URL or QR code to access detailed project information and to provide digital informed consent, after which they responded to an online survey. Consenting students who had provided their email addresses were contacted again and asked to complete the web survey at 5 and 10 months postbaseline. Respondents received no compensation.

The three time points included in the current study roughly correspond to the first three waves of the pandemic in Sweden. The Swedish Public Health Agency^21^ reports that COVID-19 was introduced in early February 2020 with a steady increase in infection during that spring, followed by further intense waves during the winter of 2020–2021, and the spring of 2021. This was followed by another intense wave the following winter, after which the scope of the pandemic slowly diminished.

In Sweden, there are 31 public HEIs and around 380 000 students. At baseline, 4497 consents were recorded from students at 19 HEIs. Of these, 3125 (69.5%) consented to participate in follow-ups, which yielded 2796 (89.5) unique email addresses and thus correspond to the sample size. Online supplemental tables 1 and 2 include the exact number of included participants at different time points (ie, column: total count). A detailed description of the initial participants is provided by Berman *et al*.^22^ The participants were students from different educational programmes (ie, different subject areas). In comparison to the average student, mean age compares well, while women were slightly overrepresented.

10.1136/bmjopen-2023-077396.supp1 Supplementary data

10.1136/bmjopen-2023-077396.supp7 Supplementary data

10.1136/bmjopen-2023-077396.supp8 Supplementary data

### Measures

The self-report online survey used in the current study was constructed to systematically research any changes taking place during the initial—most challenging—period of the pandemic. The development of the survey is described in Berman *et al*,^22^ and the complete survey can be found in a previous publication from our research group^23^ and in our preregistered analysis plan.^24^ The survey covers five areas: (a) demographics; (b) behaviours relating to COVID-19 (extent of following recommendations; if not followed, why not); (c) personal experiences of COVID-19 symptoms; (d) mental health effects of the pandemic and (e) effects on respondents’ academic self-efficacy, and satisfaction with university’s management of the pandemic. The three constructs in focus within the current study were assessed through single-item questions as follows:

1. Contagion in a cohabitant, that is, someone living with the respondent, at baseline and in a cohabitant at the 5-month follow-up, as well as contagion in family members not living with the respondent at baseline and at the 5-month follow-up. The respondent was asked to choose one of the following six response alternatives: ‘no symptoms’, ‘mild symptoms’, ‘moderate symptoms’, ‘severe symptoms’, ‘died’ and ‘not relevant/do not know’.
2. Mental health was assessed with the following question ‘How has your mental health been affected by the COVID-19 pandemic during the past 4 weeks?’. The respondent was asked to choose one of the following four response alternatives: ‘no effect’, ‘my mental health has been worse’, ‘my mental health has been better’ and ‘my mental health has been both better and worse’.
3. Academic self-efficacy was assessed with the question ‘How have your studies been going during the past 4 weeks?’. The respondent was asked to choose one of the following five response alternatives: ‘no change, my studies are going as usual’, ‘my studies have been going worse’, ‘my studies have been going better’, ‘my studies have been going both better and worse (in differing ways)’ and ‘I am not studying at this time’.

### Analysis

The complete analysis plan for this longitudinal study is available through the Open Science Framework.^24^ For this study, the estimands of interest were effects of baseline contagion in cohabitants and family members on mental health and academic self-efficacy at 5-month and 10-month follow-ups and effects of 5-month contagion in cohabitants and family members on mental health and academic self-efficacy at the 10-month follow-up. To estimate effects from observational data, a causal graph^24^ was used in conjunction with Pearl’s do-calculus^25^ to identify adjustment variables. The causal graph is presented in online supplemental figure 1 and accounts for the remaining variables at baseline and follow-ups. For each of the estimands concerning the effects of baseline factors on outcomes at 5 months, models were adjusted for baseline self-reported symptoms of COVID-19 contagion in cohabitants, family members, acquaintances, other individuals whom the respondent had been in contact with or knew of, as well as compliance with public health recommendations. For estimands concerning the effects of 5-month factors on outcomes at 10 months, models were adjusted for the same variables but measured at the 5-month follow-up. Included in the analysis are response data, that is, missing data analysis.

10.1136/bmjopen-2023-077396.supp2 Supplementary data

Multilevel multinomial regression was used to estimate effects, with adaptive intercepts for universities and subjects in longitudinal models. Models were estimated using Bayesian inference.^26^ Covariates and adaptive intercepts were given standard normal priors, and posterior distributions were used as point estimates of effects, alongside 2.5% and 97.5% posterior percentiles representing a 95% compatibility interval (CI). The posterior probability of effect estimates being less than or greater than the null is also reported.

### Patient and public involvement

None.

## Results

In online supplemental table 1, the distributions of self-reports regarding the contagion of COVID-19 in someone living with the respondent and in a family member, at baseline and 5 months after the baseline assessment, are cross-tabulated with the self-reported change in mental health at the 5-month and 10-month follow-ups. Online supplemental table 2 shows the corresponding cross-tabulations for the change in academic self-efficacy.

In relation to contagion in both someone living with the respondent and in a family member, and at all time points, ‘no symptoms’ was the most frequent response, followed by ‘don’t know’, ‘mild symptoms’, ‘moderate symptoms’, ‘severe symptoms’ and finally, ‘death’. As shown in online supplemental table 1, ‘worse’ mental health was the most frequent response at both follow-ups. At the 5-month follow-up, ‘no change’ was the second most frequent response, followed by ‘both’, while this order was the opposite at the 10-month follow-up. As displayed in online supplemental table 2, ‘worse’ academic self-efficacy was the most frequent response, followed by ‘both’ at the 5-month follow-up, while this order was the opposite at the 10-month follow-up.

### Did early contagion in cohabitants and family members affect mental health?

**Figure 1:**
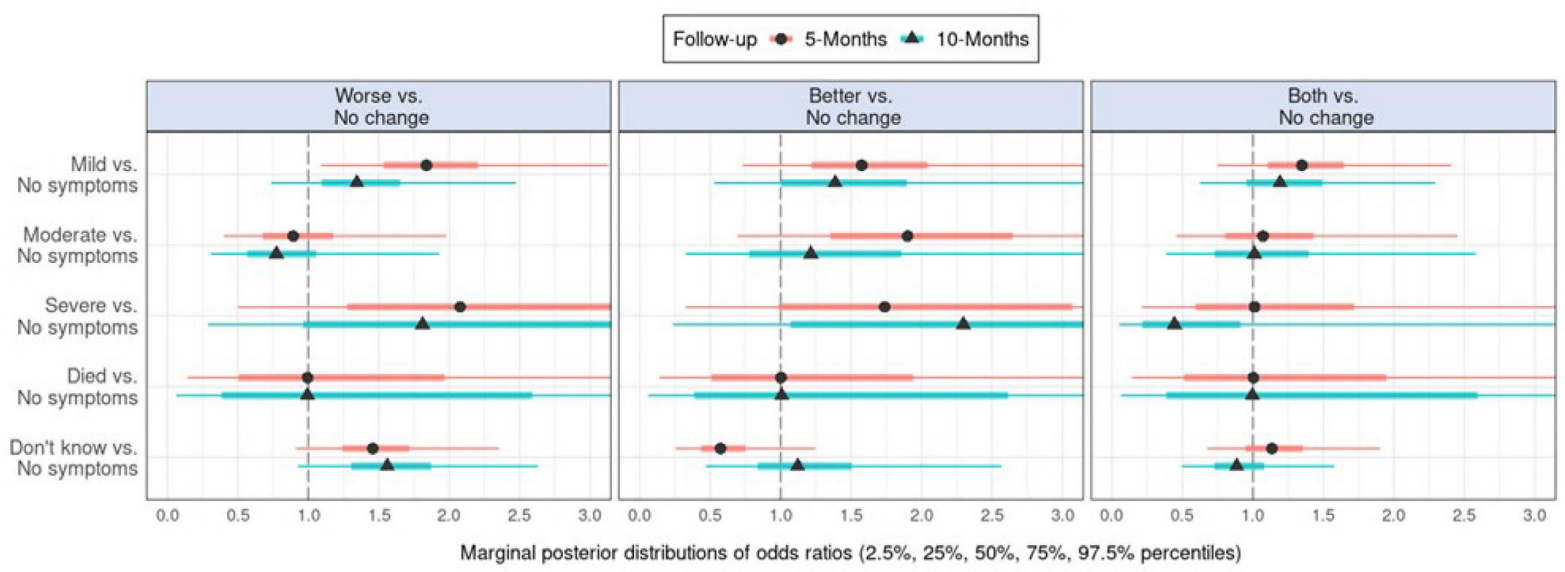
Marginal posterior distributions of coefficients in the multinomial regression models estimating effects of contagion in someone living with the respondent at baseline on self-reported change in mental health at 5-month and 10-month follow-ups.

Figure 1 presents marginal posterior distributions of coefficients in the multilevel multinomial regression models, estimating the effects of baseline contagion in cohabitants on self-reported change in mental health at 5-month and 10-month follow-ups. Online supplemental figure 2 shows the corresponding effect estimates of 5-month postbaseline contagion in cohabitants on change in mental health at the 10-month follow-up. Online supplemental table 3 provides numerical details.

10.1136/bmjopen-2023-077396.supp3 Supplementary data

10.1136/bmjopen-2023-077396.supp9 Supplementary data

As shown in figure 1, mild symptoms in cohabitants at baseline were associated with increased odds of reporting worse mental health rather than no change in mental health at the 5-month follow-up. However, the findings suggest that the effect had waned at 10 months. There was no marked evidence of effects of symptoms at 5 months postbaseline, on mental health at 10 months (online supplemental figure 2). Across all estimates, there was considerable uncertainty regarding the effects of symptoms of contagion in cohabitants.

Online supplemental figure 3 and 4 show the corresponding effect estimates of contagion in family members, with online supplemental table 4 providing numerical details. The estimates suggest that self-reports of symptoms of contagion in family members at baseline and at 5 months had no effect on mental health at any of the follow-ups. Again, there was considerable uncertainty in all estimates.

10.1136/bmjopen-2023-077396.supp4 Supplementary data

10.1136/bmjopen-2023-077396.supp5 Supplementary data

10.1136/bmjopen-2023-077396.supp10 Supplementary data

### Did contagion in cohabitants and family members affect academic self-efficacy?

**Figure 2:**
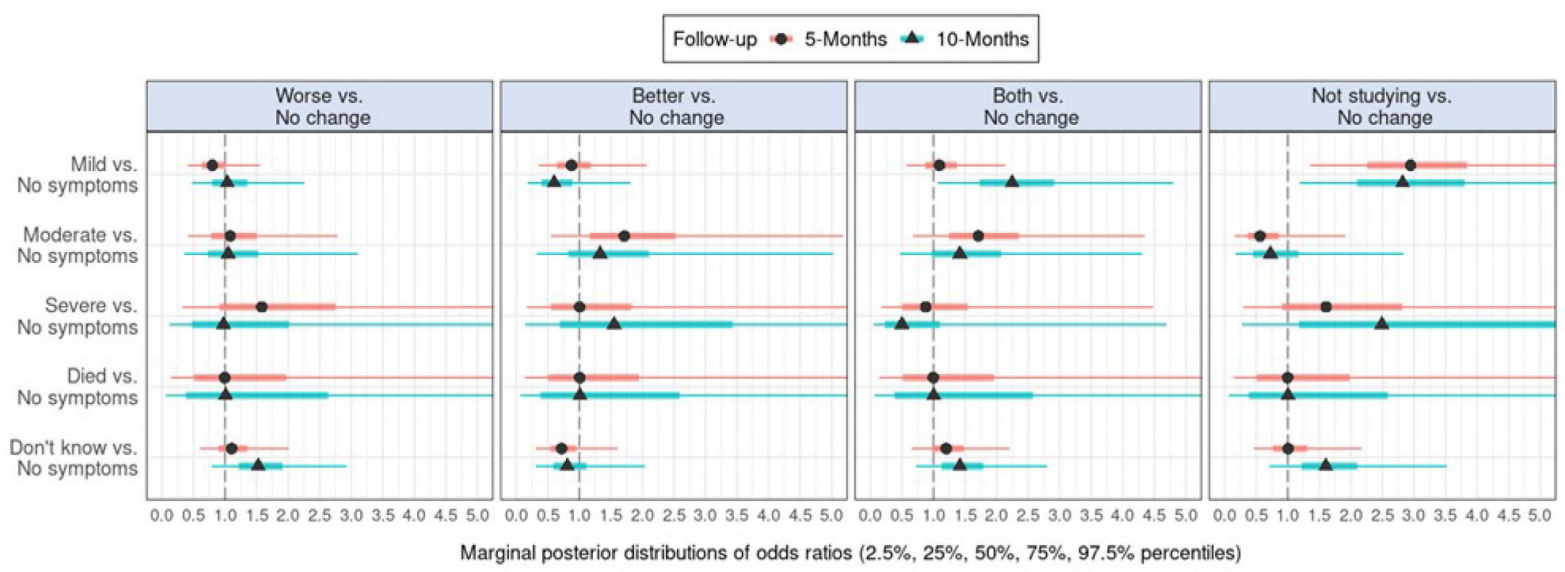
Marginal posterior distributions of coefficients in the multinomial regression models estimating effects of contagion in someone living with the respondent at baseline on self-reported change in academic self-efficacy at 5-month and 10-month follow-ups.

**Figure 3:**
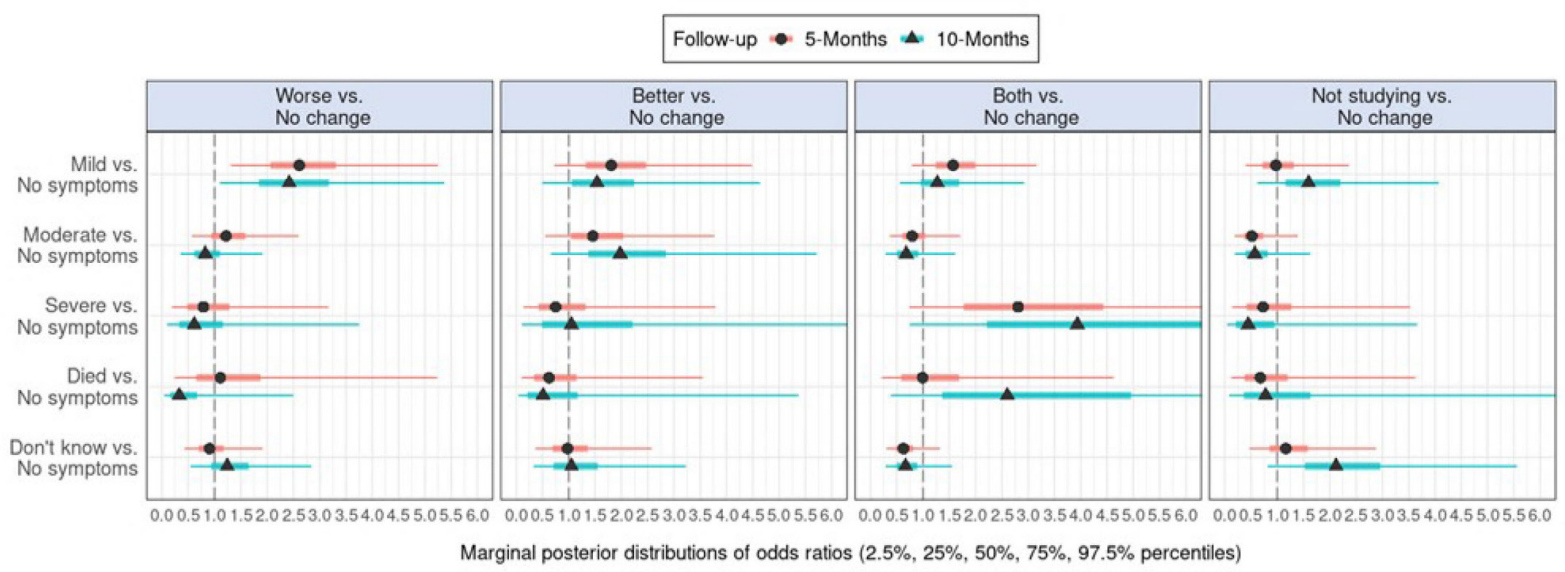
Marginal posterior distributions of coefficients in the multinomial regression models estimating effects of contagion in family member at baseline on self-reported change in academic self-efficacy at 5-month and 10-month follow-ups.

Following the same analytical procedure, figure 2 and online supplemental figure 5 show effect estimates on contagion in cohabitants in relation to academic self-efficacy. Figure 3 and online supplemental figure 6 show estimates of contagion in family members in relation to academic self-efficacy. Online supplemental table 5 shows numerical details regarding cohabitants while online supplemental table 6 shows numerical details regarding family members.

10.1136/bmjopen-2023-077396.supp6 Supplementary data

10.1136/bmjopen-2023-077396.supp12 Supplementary data

10.1136/bmjopen-2023-077396.supp13 Supplementary data

As shown in figure 2, effect estimates indicate that mild symptoms in cohabitants at baseline were associated with increased odds of reporting not studying, rather than no change in academic self-efficacy at both 5-month and 10-month follow-ups.

In figure 3, effect estimates show that mild symptoms of contagion in a family member at baseline were associated with increased odds of reporting worse academic self-efficacy rather than no change at both 5-month and 10-month follow-ups.

## Discussion

The current study reports that the contagion of COVID-19 in cohabitants and family members has led to some negative effects on both mental health and academic self-efficacy in university students in Sweden. Mild baseline symptoms reported in cohabitants in May 2020 resulted in negative mental health effects 5 months later, and mild baseline symptoms in family members resulted in negative effects on academic self-efficacy both 5 and 10 months later.

The more prolonged effects of baseline contagion related to family members can probably be explained by an awareness of the increased risk contagion in relation to older people as compared with younger^27^; this reasoning assumes, however, that cohabitants (ie, partners, fellow students) were younger than family members (ie, parents). The patterns relating to mild symptoms in the early phase of the pandemic were not found in the second stage when the effects of 5-month contagion in cohabitants and family members on student mental health were assessed 10 months postbaseline. A potential explanation for not identifying any effect in the second stage may relate to students having learnt to cope with mild symptoms of contagion seen in cohabitants and family members after an initial period of stress caused by the unfamiliar condition.^28^ The lack of effects on mental health in students reporting more severe symptoms or deaths might be attributed to small sample sizes in the groups of students reporting more severe symptoms in both cohabitants and family members.

Mild baseline symptoms of contagion in cohabitants were also related to increased odds of reporting not studying rather than no change in academic self-efficacy at both follow-ups. It remains unclear whether the non-studying category included students who had left higher education due to the pandemic, or by students having finalised their studies, or any other reason. However, it seems reasonable that those not studying at the follow-ups reported low academic self-efficacy.

Social relationships and the health of cohabitants and family members are considered key in relation to mental health^2 4^ and have been identified as essential in relation to mental health and academic self-efficacy in university students.^6 7^ While the current findings differ from a previous cross-sectional finding from Sweden, where no marked associations were identified between symptoms of contagion in different social contexts and student mental health and study capacity,^16^ the findings do align with cross-sectional studies from the USA and Spain.^14 15^ Thus, the current longitudinal study expands previous findings from Sweden and adds information on factors contributing to mental health problems and academic self-efficacy during a longer crisis.

University students have been identified as a vulnerable group, with a high prevalence of mental disorders and suicidal thoughts.^5^ Research also shows the importance of social relationships as many students experiencing problems prefer to talk to friends or family instead of seeking professional care.^29^ The current findings emphasise the need to provide students with sufficient support in times of crisis and university lockdown, which limit access to the social arena that the university provides. According to the law, students at Swedish HEIs have access to student health services, which complement the general healthcare system in having a stronger focus on studies and preventive measures. During the pandemic, mental health services were rapidly transferred to online counselling. To prepare student health services for upcoming crises, it is key to identify factors that impact student mental health and academic self-efficacy. A recent qualitative study examining student perspectives on pandemic-induced intervention needs suggests that universities should consider introducing routine screening and digital interventions.^30^ Considering that university students in general seem willing to participate in sensitive health interventions through digital screening^31^ and that digital interventions targeting multiple health problems relevant to the student population are being developed,^32^ this may be an effective solution. Moreover, introducing peer-led mental health interventions offers several advantages.^33^ These include increased accessibility and relatability, as peers can understand and empathise with the unique challenges of a crisis. In contrast to the emergency remote teaching typically launched during the pandemic, further development of sustainable online learning platforms that promote social interaction would be valuable for any future crises but also to facilitate collaboration and online learning.^34^

### Strengths

There are few prospective longitudinal studies of the effects of COVID-19 on university students, and this is a unique study focusing on early COVID-19 contagion in family members and its effects on academic self-efficacy and mental health among students in Sweden. The methodological strengths of our study include its longitudinal design and the use of a robust approach to defining causality. Using a causal model allows for examining the effects of observational data, based on the assumptions conveyed in a predefined graph.^24^ This, in turn, informs the use of do-calculus to determine which variables should be adjusted to avoid confounding. Based on the causal model, estimand analyses were adjusted for contagion in the social network and compliance with public health recommendations. Some might argue that the present analysis should be adjusted for self-contagion. Here, however, self-contagion is placed downstream in the causal model and is thus considered to mediate the effects of contagion in cohabitants and family members on mental health and academic self-efficacy.

### Limitations

The use of a causal graph means that the findings are limited by the graph’s representativeness of actual causal relationships. Thus, if they do not hold, then the estimates of effects are potentially still confounded and should be interpreted as associations. There is no protection against spurious associations induced by collider bias, apart from where time can guarantee the direction of causality. This means that the estimates presented in the analysis should only be understood to represent effects if the causal model is appropriate.

The limited sample size of the current study in relation to the number of students reporting severe symptoms or deaths in cohabitants and family members was fortunately low. Still, this obviously limits the possibility of fully exploring the effects of negative consequences other than self-reported mild symptoms in social relationships, in this case in cohabitants and family members. Moreover, it should be noted that this study includes a limited group of students, from a limited number of universities, who volunteered their participation after responding to an advertisement. These circumstances mean that the participants might not be representative of the total group of students, an issue discussed in a previous publication from our research group.^22^ An additional limitation could also be that findings may have been affected by non-response vis-à-vis the two follow-ups. There is one final limitation to consider, and it involves the use of single items. In comparison to multiple-item measures, single items are generally considered to have lower content validity, sensitivity and reliability. However, single-item questions can still be used as a substitute for multiple-item measures, especially when it comes to capturing global phenomena. For instance, when participants are asked to rate their overall health with a single question, it tends to align with the overall scores obtained from multiple-item measures.^35^ Moreover, single-item questions have the added advantage of reducing the response burden for participants.

## Conclusions

The current study shows that mild contagion in cohabitants and family members during COVID-19 had negative longitudinal effects on the mental health and academic self-efficacy of university students in Sweden. The findings emphasise the importance of social relationships and the challenges involved in providing students with sufficient support in times of crisis. Universities need to pay attention to the fact that students are influenced by events in their social networks and be prepared to support student mental health and academic self-efficacy. Finally, we note that this study contributes to the Global Goals of the 2030 Agenda for Sustainable Development by providing longitudinal findings and insights that can inform policies and interventions aimed at promoting mental health, supporting students and addressing the challenges related to external factors during crises such as the COVID-19 pandemic.

10.1136/bmjopen-2023-077396.supp11 Supplementary data

## Data Availability

Data are available on reasonable request.
