# Supplementary material for: Effects of COVID-19 contagion in cohabitants and family members on mental health and academic self-efficacy among university students in Sweden: a prospective longitudinal study
Source: BMJ Open. 2024 Mar 12;14(3):e077396. doi: 10.1136/bmjopen-2023-077396 (PMC10936505; doi:10.1136/bmjopen-2023-077396)
Supplement: Supplementary data [file bmjopen-2023-077396supp001.pdf]

SUPPLEMENTARY FIGURE LEGENDS

Supplementary Figure 1. Causal Model. Causal assumptions made to estimate effects from observational data. Abbreviations: The letter B refers to Baseline data. M5 and M10 refer to the two follow-up assessments. Acad. Self-efficacy means Academic Self-Efficacy; Sit. means Situation; Symp. means Symptoms; Uni. Man. Pand. means Universities Management of the Pandemic. As part of the statistical analysis plan, a complete list of included variables can be viewed and downloaded from OSF [24]. To enhance the readability, the figure can be enlarged in the digital version of this publication.

Supplementary Figure 2. Marginal posterior distributions of coefficients in the multinomial regression models estimating effects of contagion in someone living with the respondent at 5-months follow-up on self-reported change in mental health at 10-months follow-up.

Supplementary Figure 3. Marginal posterior distributions of coefficients in the multinomial regression models estimating effects of contagion in family member at baseline on self-reported change in mental health at 5-months and 10-months follow-ups.

Supplementary Figure 4. Marginal posterior distributions of coefficients in the multinomial regression models estimating effects of contagion in family member at 5-months follow-up on self-reported change in mental health at 10-months follow-up.

Supplementary Figure 5. Marginal posterior distributions of coefficients in the multinomial regression models estimating effects of contagion in someone living with the respondent at 5-months follow-up on self-reported change academic self-efficacy at 10-months follow-up.

Supplementary Figure 6. Marginal posterior distributions of coefficients in the multinomial regression models estimating effects of contagion in family member at 5-months follow-up on self-reported change in academic self-efficacy at 10-months follow-up.

SUPPLEMENTARY TABLE LEGENDS

Supplementary Table 1. Contingency table (frequency) showing the distribution of self-reported symptoms of COVID-19 contagion in individuals living with the respondent and their family members at baseline and five months after the baseline assessment, in relation to self-reported change in mental health at the 5-month and 10-month follow-ups.

Supplementary Table 2. Contingency table (frequency) showing the distribution of self-reported symptoms of COVID-19 contagion in individuals living with the respondent and their family members at baseline and five months after the baseline assessment, in relation to self-reported change in academic self-efficacy health at the 5-month and 10-month follow-ups.

Andersson C, *et al. BMJ Open* 2024; 14:e077396. doi: 10.1136/bmjopen-2023-077396

Supplementary Table 3. Contagion in someone living with the respondent at baseline and at 5-month follow-up in relation to self-reported change in mental health at 5-month and 10-month follow-ups. Reported are medians of the marginal posterior distributions of odds ratios with 2.5% and 97.5% percentiles, followed by posterior probability that the odds ratio is greater or less than 1 (in direction of the median).

Supplementary Table 4. Contagion in family members at baseline and at 5-month follow-up in relation to self-reported change in mental health at 5-month and 10-month follow-ups. Reported are medians of the marginal posterior distributions of odds ratios with 2.5% and 97.5% percentiles, followed by posterior probability that the odds ratio is greater or less than 1 (in direction of the median).

Supplementary Table 5. Contagion in someone living with the respondent at baseline and at 5-month follow-up in relation to self-reported change in academic self-efficacy at 5-month and 10-month follow-ups. Reported are medians of the marginal posterior distributions of odds ratios with 2.5% and 97.5% percentiles, followed by posterior probability that the odds ratio is greater or less than 1 (in direction of the median).

Supplementary Table 6. Contagion in family member at baseline and at 5-month follow-up in relation to self-reported change in academic self-efficacy at 5-month and 10-month follow-ups. Reported are medians of the marginal posterior distributions of odds ratios with 2.5% and 97.5% percentiles, followed by posterior probability that the odds ratio is greater or less than 1 (in direction of the median).

Andersson C, *et al. BMJ Open* 2024; 14:e077396. doi: 10.1136/bmjopen-2023-077396
