# Supplementary figures and images for: Effects of COVID-19 contagion in cohabitants and family members on mental health and academic self-efficacy among university students in Sweden: a prospective longitudinal study
Source: BMJ Open. 2024 Mar 12;14(3):e077396. doi: 10.1136/bmjopen-2023-077396 (PMC10936505; doi:10.1136/bmjopen-2023-077396)

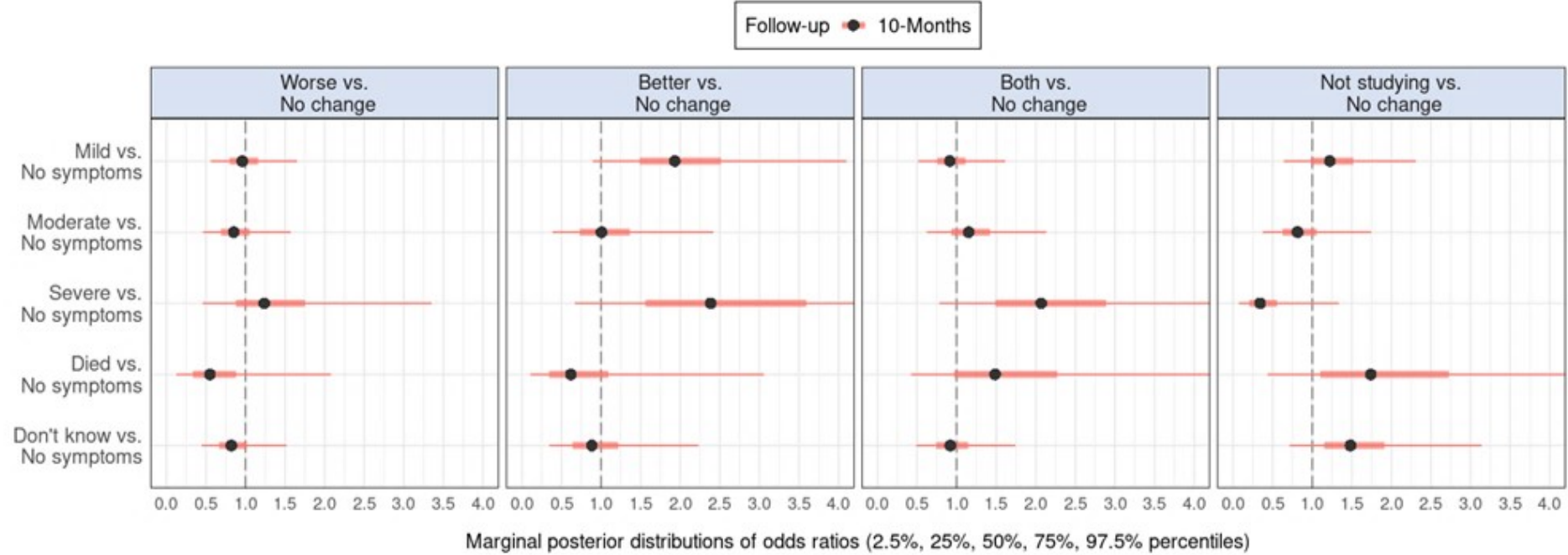

Supplement: Supplementary data [file bmjopen-2023-077396supp007.pdf]

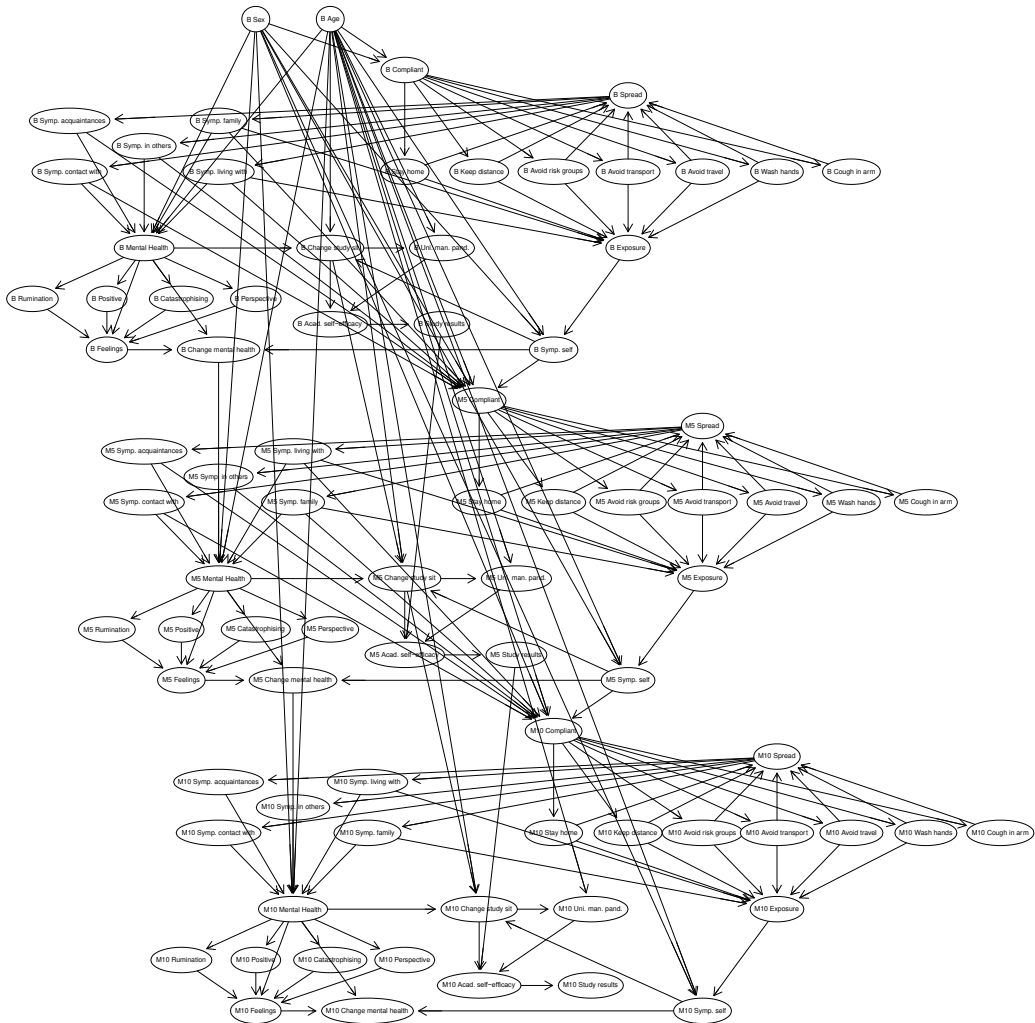

Supplement: Supplementary data [file bmjopen-2023-077396supp002.pdf]

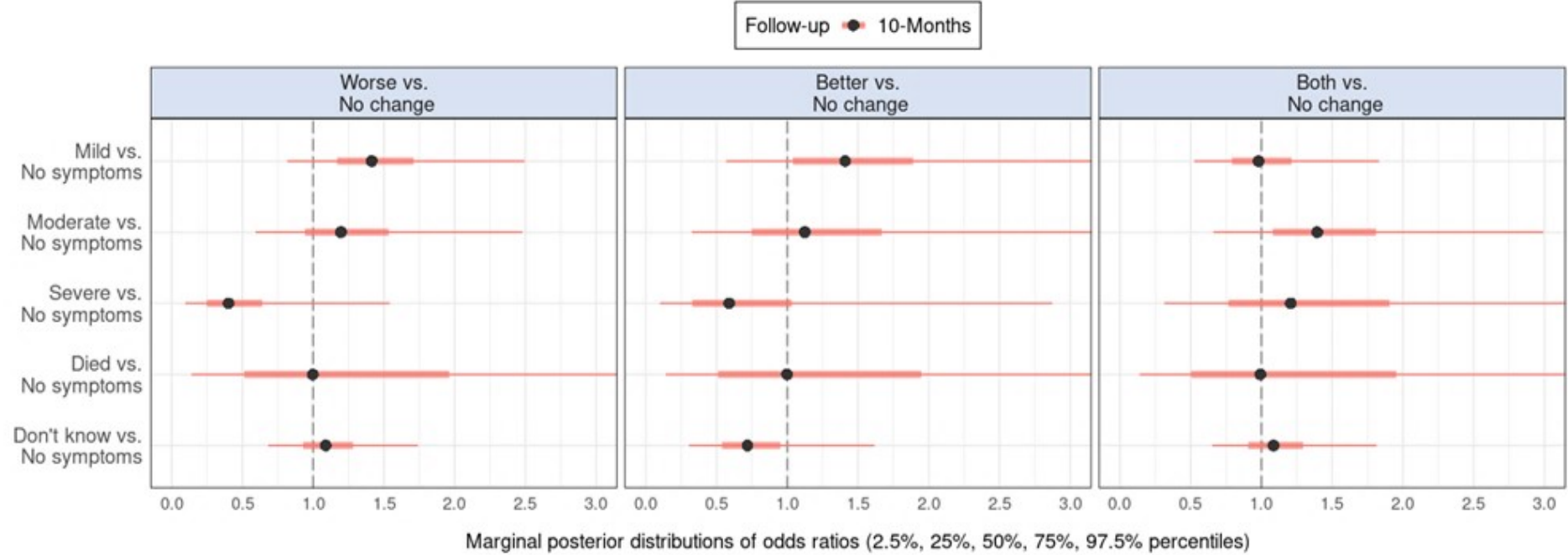

Supplement: Supplementary data [file bmjopen-2023-077396supp003.pdf]

Follow-up ● 5-Months ▲ 10-Months

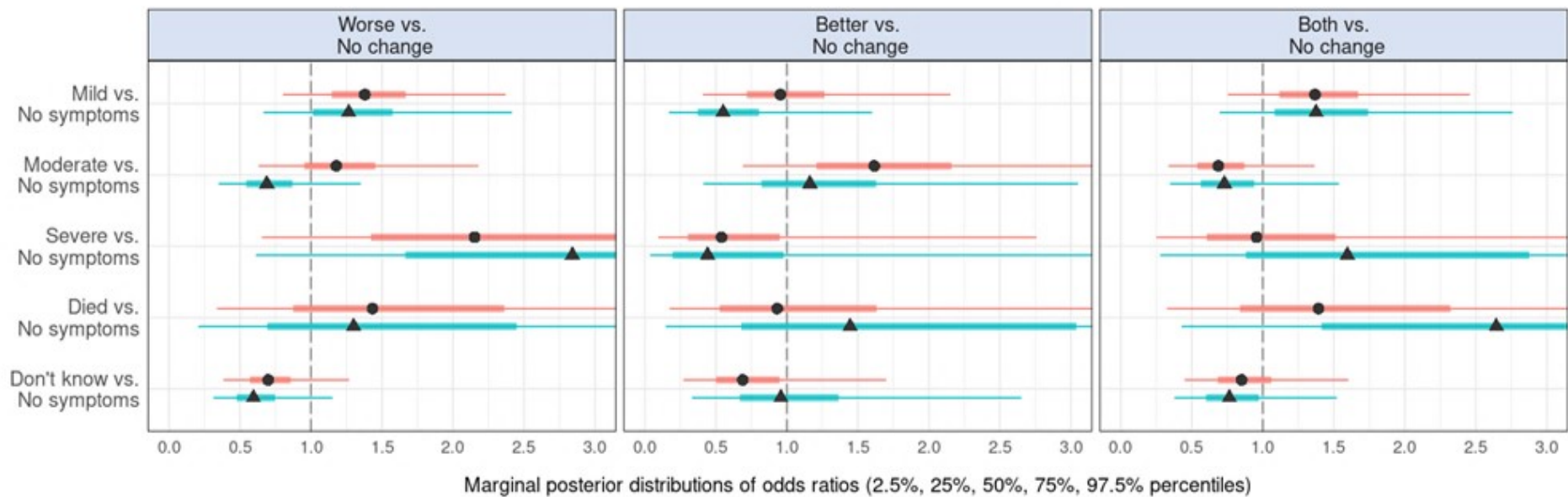

Supplement: Supplementary data [file bmjopen-2023-077396supp004.pdf]

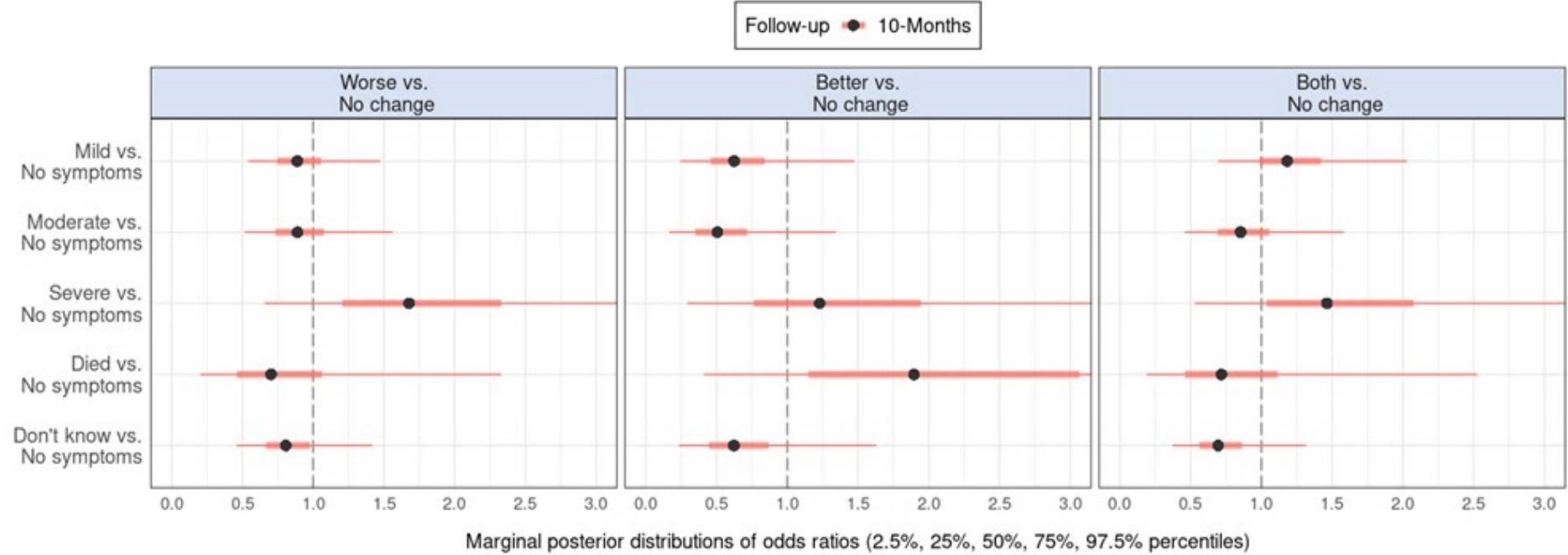

Supplement: Supplementary data [file bmjopen-2023-077396supp005.pdf]

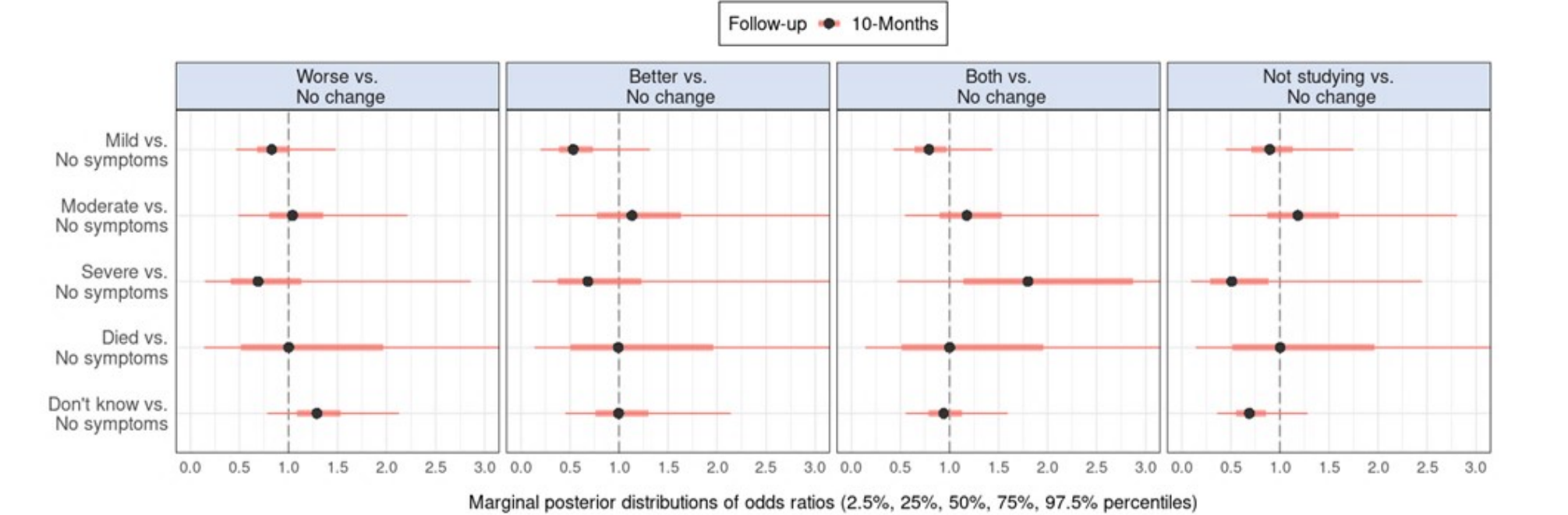

Supplement: Supplementary data [file bmjopen-2023-077396supp006.pdf]
