# Supplementary material for: Effects of COVID-19 contagion in cohabitants and family members on mental health and academic self-efficacy among university students in Sweden: a prospective longitudinal study
Source: BMJ Open. 2024 Mar 12;14(3):e077396. doi: 10.1136/bmjopen-2023-077396 (PMC10936505; doi:10.1136/bmjopen-2023-077396)
Supplement: Supplementary data [file bmjopen-2023-077396supp008.pdf]

Supplementary Table 1. Contingency table (frequency) showing the distribution of self-reported symptoms of COVID-19 contagion in individuals living with the respondent and their family members at baseline and five months after the baseline assessment, in relation to self-reported change in mental health at the 5-month and 10-month follow-ups.

		Self-reported change in mental health									
		5-month follow-up					10-month follow-up				
		No change	Worse	Better	Both	Total	No change	Worse	Better	Both	Total
Self-reported symptoms of Covid-19 contagion in somebody living with the respondent at baseline	No symptoms	359	315	56	221	951	206	372	56	232	866
	Mild symptoms	63	90	17	54	224	37	91	12	55	195
	Moderate symptoms	25	28	12	18	83	18	30	6	25	79
	Severe symptoms	1	8	2	2	13	2	5	1	3	11
	Died	0	0	0	0	0	0	0	0	0	0
	Don't know	145	164	14	93	416	81	181	17	93	372
	Total	593	605	101	388	1687	344	679	92	408	1523
Self-reported symptoms of Covid-19 contagion in someone living with the respondent at five months post-baseline	No symptoms						192	326	45	202	765
	Mild symptoms						28	77	11	41	157
	Moderate symptoms						13	35	3	21	72
	Severe symptoms						3	1	0	3	7
	Died						0	0	0	0	0
	Don't know						83	166	14	95	358
	Total						319	605	73	362	1359
Self-reported symptoms of Covid-19 contagion in a family member at baseline	No symptoms	365	347	62	236	1010	201	411	57	239	908
	Mild symptoms	66	84	13	53	216	37	85	7	53	182
	Moderate symptoms	53	66	11	31	161	40	69	12	37	158
	Severe symptoms	7	16	2	6	31	5	14	4	8	31
	Died	4	3	0	2	9	1	4	1	5	11
	Don't know	104	96	12	60	272	64	98	11	66	239
	Total	599	612	100	388	1699	348	681	92	408	1529
Self-reported symptoms of Covid-19 contagion in a family member at five months post-baseline	No symptoms						182	339	48	203	772
	Mild symptoms						42	88	7	56	193
	Moderate symptoms						35	57	7	40	139
	Severe symptoms						4	14	2	10	30
	Died						3	5	2	2	12
	Don't know						55	105	10	56	226
	Total						321	608	76	367	1372
