# Supplementary material for: Effects of COVID-19 contagion in cohabitants and family members on mental health and academic self-efficacy among university students in Sweden: a prospective longitudinal study
Source: BMJ Open. 2024 Mar 12;14(3):e077396. doi: 10.1136/bmjopen-2023-077396 (PMC10936505; doi:10.1136/bmjopen-2023-077396)
Supplement: Supplementary data [file bmjopen-2023-077396supp009.pdf]

Supplementary Table 2. Contingency table (frequency) showing the distribution of self-reported symptoms of COVID-19 contagion in individuals living with the respondent and their family members at baseline and five months after the baseline assessment, in relation to self-reported change in academic self-efficacy health at the 5-month and 10-month follow-ups.

		Self-reported change in academic self-efficacy											
		5-month follow-up						10-month follow-up					
		No change	Worse	Better	Both	Not studying	Total	No change	Worse	Better	Both	Not studying	Total
Self-reported symptoms of Covid-19 contagion in somebody living with the respondent at baseline	No symptoms	195	293	63	302	84	937	187	266	58	237	112	860
	Mild symptoms	40	62	17	69	35	223	27	53	12	65	35	192
	Moderate symptoms	15	26	7	29	5	82	16	23	8	20	11	78
	Severe symptoms	0	9	0	3	1	13	2	4	1	2	2	11
	Died	0	0	0	0	0	0	0	0	0	0	0	0
	Don’t know	84	137	23	132	37	413	68	124	23	96	59	370
	Total	334	527	110	535	162	1668	300	470	102	420	219	1511
Self-reported symptoms of Covid-19 contagion in someone living with the respondent at five months post-baseline	No symptoms							157	232	55	200	115	759
	Mild symptoms							31	52	6	42	23	154
	Moderate symptoms							11	22	5	18	16	72
	Severe symptoms							2	1	0	4	0	7
	Died							0	0	0	0	0	0
	Don’t know							71	120	21	102	43	357
	TOTAL							272	427	87	366	197	1349
Self-reported symptoms of Covid-19 contagion in a family member at baseline	No symptoms	213	300	61	320	103	997	190	281	54	259	119	903
	Mild symptoms	23	81	21	71	19	215	28	62	12	48	30	180
	Moderate symptoms	29	59	10	49	12	159	33	45	15	42	21	156
	Severe symptoms	6	7	1	15	2	31	4	8	4	11	3	30
	Died	3	5	0	1	0	9	3	1	0	5	2	11
	Don’t know	59	82	16	84	28	269	44	78	15	56	44	237
	Total	333	534	109	540	164	1680	302	475	100	421	219	1517
Self-reported symptoms of Covid-19 contagion in a family member at five months post-baseline	No symptoms							157	248	48	207	107	767
	Mild symptoms							39	58	15	52	29	193
	Moderate symptoms							27	44	10	34	24	139
	Severe symptoms							4	9	4	12	1	30
	Died							3	1	0	5	3	12
	Don’t know							41	70	12	61	38	222
	Total							271	430	89	371	202	1363
