# Supplementary material for: Effects of COVID-19 contagion in cohabitants and family members on mental health and academic self-efficacy among university students in Sweden: a prospective longitudinal study
Source: BMJ Open. 2024 Mar 12;14(3):e077396. doi: 10.1136/bmjopen-2023-077396 (PMC10936505; doi:10.1136/bmjopen-2023-077396)
Supplement: Supplementary data [file bmjopen-2023-077396supp010.pdf]

Supplementary Table 3. Contagion in someone living with the respondent at baseline and at 5-months follow-up in relation to self-reported change in mental health at 5-months and 10-months follow-ups. Reported are medians of the marginal posterior distributions of odds ratios with 2.5% and 97.5% percentiles, followed by posterior probability that the odds ratio is greater or less than 1 (in direction of the median).

		Self-reported change in mental health					
		5-months follow-up			10-months follow-up		
		Worse vs. No change	Better vs. No change	Both vs. No change	Worse vs. No change	Better vs. No change	Both vs. No Change
Self-reported symptoms of Covid-19 contagion in somebody living with the respondent at baseline	Mild vs No symptoms	1.84 (1.09; 3.12)/98.9%	1.58 (0.73; 3.29)/88.3%	1.35 (0.75; 2.41)/84.4%	1.34 (0.74; 2.47)/83.3%	1.39 (0.53; 3.41)/75.3%	1.19 (0.63; 2.29)/70.1%
	Moderate vs No symptoms	0.89 (0.40; 1.98)/60.9%	1.90 (0.69; 4.99)/89.6%	1.07 (0.46; 2.45)/56.4%	0.77 (0.31; 1.93)/71.0%	1.21 (0.33; 4.08)/61.6%	1.01 (0.39; 2.58)/50.9%
	Severe vs No symptoms	2.08 (0.5; 8.64)/84.3%	1.74 (0.33; 9.85)/74.4%	1.01 (0.21; 4.62)/50.6%	1.81 (0.29; 11.48)/73.7%	2.30 (0.24; 19.58)/76.8%	0.44 (0.05; 3.50/77.7%
	Died vs No symptoms	1.00 (0.14; 7.00)/50.2%	1.00 (1.14; 7.10)/50.1%	1.00 (0.14; 7.01)/50.1%	0.99 (0.06; 16.38)/50.1%	1.01 (0.06; 15.89)/50.2%	1.00 (0.06; 15.63)/50.1%
	Don’t know vs No symptoms	1.46 (0.91; 2.35)/94.3%	0.57 (0.26; 1.25)/92.1%	1.13 (0.68; 1.90)/60.3%	1.56 (0.93; 2.63)/95.2%	1.12 (0.47; 2.57)/60.5%	0.89 (0.49; 1.58)/66.1%
Self-reported symptoms of Covid-19 contagion in somebody living with the respondent at five months post-baseline	Mild vs No symptoms				1.41 (0.82; 2.49)/89.0%	1.41 (0.57; 3.27)/77.6%	0.98 (0.53; 1.83)/52.5%
	Moderate vs No symptoms				1.20 (0.59; 2.48)/69.2%	1.12 (0.33; 3.41)/57.7%	1.39 (0.66; 2.99)/80.9%
	Severe vs No symptoms				0.40 (0.10; 1.54)/90.6%	0.59 (0.10; 2.87)/73.9%	1.21 (0.31; 4.60)/60.9%
	Died vs No symptoms				1.00 (0.14; 7.12)/50.1%	1.00 (0.14; 7.16)/50.1%	0.99 (0.14; 7.24)/50.3%
	Don’t know vs No symptoms				1.09 /0.68; 1.74)/64.1%	0.72 (0.30; 1.62)/78.5%	1.09 (0.65; 1.81)/62.1%
