# Supplementary material for: Effects of COVID-19 contagion in cohabitants and family members on mental health and academic self-efficacy among university students in Sweden: a prospective longitudinal study
Source: BMJ Open. 2024 Mar 12;14(3):e077396. doi: 10.1136/bmjopen-2023-077396 (PMC10936505; doi:10.1136/bmjopen-2023-077396)
Supplement: Supplementary data [file bmjopen-2023-077396supp012.pdf]

BMJ Publishing Group Limited (BMJ) disclaims all liability and responsibility arising from any reliance placed on this supplemental material which has been supplied by the author(s)

Supplementary Table 5. Contagion in someone living with the respondent at baseline and at 5-months follow-up in relation to self-reported change in academic self-efficacy at 5-months and 10-months follow-ups. Reported are medians of the marginal posterior distributions of odds ratios with 2.5% and 97.5% percentiles, followed by posterior probability that the odds ratio is greater or less than 1 (in direction of the median).

		Self-reported change in academic self-efficacy							
		5-months follow-up				10-months follow-up			
		Worse vs. No change	Better vs. No change	Both vs. No change	Not studying vs. No change	Worse vs. No change	Better vs. No change	Both vs. No Change	Not studying vs. No change
Self-reported symptoms of Covid-19 contagion in somebody living with the respondent at baseline	Mild vs No symptoms	0.80 (0.41; 1.55)/74.4%	0.88 (0.35; 2.07)/61.8%	1.09 (0.57; 2.14)/60.2%	2.95 (1.36; 6.38)/99.7%	1.03 (0.47; 2.25)/53.4%	0.60 (0.18; 1.81)/81.1%	2.25 (1.06; 4.80)/98.3%	2.82 (1.19; 6.74)/99.1
	Moderate vs No symptoms	1.08 (0.41; 2.78)/56.5%	1.71 (0.55; 5.18)/82.1%	1.71 (0.67; 4.34)/87.2%	0.56 (0.16; 1.91)/82.2%	1.05 (0.36; 3.10)/53.7%	1.33 (0.32; 5.02)/65.4%	1.42 (0.47; 4.30)/73.1%	0.73 (0.17; 2.83)/67.5%
	Severe vs No symptoms	1.58 (0.32; 7.64)/71.4%	1.00 (0.17; 5.63)/50.2%	0.88 (0.17; 4.48)/56.4%	1.61 (0.30; 8.57)/71.1%	0.97 (0.12; 7.96)/51.0%	1.55 (0.14; 15.75)/64.2%	0.50 (0.05; 4.69)/72.4%	2.49 (0.28; 21.86)/79.2%
	Died vs No symptoms	0.99 (0.14; 7.15)/50.2%	1.01 (0.14; 7.30)/50.2%	1.00 (0.14; 7.05)/50.2%	1.00 (0.14; 7.07)/50.1%	1.01 (0.06; 16.13)/50.2%	1.01 (0.06; 15.63)/50.3%	1.00 (0.06; 15.93)/50.1%	1.00 (0.06; 15.84)/50.1%
	Don’t know vs No symptoms	1.10 (0.60; 2.00)/62.5%	0.72 (0.31; 1.61)/78.8%	1.20 (0.65; 2.21)/72.7%	1.01 (0.46; 2.17)/50.5%	1.53 (0.79; 2.93)/89.7%	0.81 (0.31; 2.04)/67.1%	1.42 (0.73; 2.80)/84.7%	1.60 (0.71; 3.52)/87.5%
Self-reported symptoms of Covid-19 contagion in somebody living with the respondent at five months post-baseline	Mild vs No symptoms					0.83 (0.47; 1.48)/73.9%	0.53 (0.20; 1.32)/90.9%	0.79 (0.43; 1.44)/77.9%	0.90 (0.45; 1.75)/62.4%
	Moderate vs No symptoms					1.04 (0.49; 2.21)/54.1%	1.13 (0.36; 3.19)/58.6%	1.18 (0.55; 2.52)/65.8%	1.18 (0.48; 2.80)/64.5%
	Severe vs No symptoms					0.69 (0.15; 2.86)/69.1%	0.68 (0.12; 3.59)/67.0%	1.80 (0.47; 6.97)/80.8%	0.51 (0.09; 2.45)/50.1%
	Died vs No symptoms					1.00 (0.14; 7.16)/50.1%	0.99 (0.14; 7.19)/50.3%	1.00 (1.14; 6.92)/50.1%	1.00 (0.14; 7.06)/50.1%
	Don’t know vs No symptoms					1.29 (0.78; 2.13)/84.0%	1.00 (0.45; 2.14)/50.5%	0.94 (0.55; 1.59)/58.9%	0.69 (0.36; 1.28)/87.6%
