# Supplementary material for: Effects of COVID-19 contagion in cohabitants and family members on mental health and academic self-efficacy among university students in Sweden: a prospective longitudinal study
Source: BMJ Open. 2024 Mar 12;14(3):e077396. doi: 10.1136/bmjopen-2023-077396 (PMC10936505; doi:10.1136/bmjopen-2023-077396)
Supplement: Supplementary data [file bmjopen-2023-077396supp013.pdf]

**Supplementary Table 6.** Contagion in family member at baseline and at 5-months follow-up in relation to self-reported change in academic self-efficacy at 5-months and 10-months follow-ups. Reported are medians of the marginal posterior distributions of odds ratios with 2.5% and 97.5% percentiles, followed by posterior probability that the odds ratio is greater or less than 1 (in direction of the median).

| | | Self-reported change in academic self-efficacy | | | | | | | |
|---|---|---|---|---|---|---|---|---|---|
| | | 5-months follow-up | | | | 10-months follow-up | | | |
| | | Worse vs. No change | Better vs. No change | Both vs. No change | Not studying vs. No change | Worse vs. No change | Better vs. No change | Both vs. No Change | Not studying vs. No change |
| Self-reported symptoms of Covid-19 contagion in a family member at baseline | Mild vs No symptoms | 2.61 (1.31; 5.25)/99.7% | 1.81 (0.72; 4.48)/89.7% | 1.57 (0.78; 3.16)/90.0% | 0.97 (0.40; 2.36)/52.5% | 2.42 (1.10; 5.37)/98.6% | 1.54 (0.50; 4.63)/77.9% | 1.27 (0.56; 2.92)/71.6% | 1.60 (0.62; 4.07)/83.6% |
| | Moderate vs No symptoms | 1.22 (0.57; 2.60)/69.6% | 1.46 (0.55; 3.77)/77.7% | 0.79 (0.37; 1.71)/72.2% | 0.52 (0.19; 1.39)/90.2% | 0.82 (0.35; 1.91)/67.2% | 1.98 (0.66; 5.71)/89.1% | 0.68 (0.29; 1.61)/81.2% | 0.57 (0.19; 1.63)/85.2% |
| | Severe vs No symptoms | 0.79 (0.19; 3.16)/62.8% | 0.75 (0.14; 3.79)/63.3% | 2.81 (0.74; 10.54)/93.5% | 0.73 (0.14; 3.53)/65.1% | 0.61 (0.09; 3.75)/69.8% | 1.05 (0.11; 8.73)/51.8% | 3.94 (0.75; 21.2)/94.7% | 0.45 (0.05; 3.66)/76.5% |
| | Died vs No symptoms | 1.11 (0.24; 5.23)/55.5% | 0.62 (0.10; 3.55)/70.0% | 0.99 (0.21; 4.62)/50.4% | 0.68 (0.12; 3.62)/67.8% | 0.33 (0.04; 2.50)/85.8% | 0.51 (0.04; 5.37)/70.8% | 2.60 (0.39; 17.62)/84.1% | 0.78 (0.08; 6.42)/58.9% |
| | Don't know vs No symptoms | 0.91 (0.43; 1.91)/60.6% | 0.97 (0.36; 2.58)/52.1% | 0.63 (0.30; 1.32)/88.9% | 1.17 (0.47; 2.88)/63.3% | 1.24 (0.54; 2.84)/69.6% | 1.05 (0.33; 3.23)/53.4% | 0.67 (0.29; 1.55)/82.5% | 2.12 (0.81; 5.56)/93.9% |
| Self-reported symptoms of Covid-19 contagion in a family member at five months post-baseline | Mild vs No symptoms | | | | | 0.96 (0.56; 1.65)/55.8% | 1.93 (0.90; 4.10)/95.3% | 0.91 (0.51; 1.61)/62.2% | 1.22 (0.64; 2.31)/73.1% |
| | Moderate vs No symptoms | | | | | 0.85 (0.46; 1.57)/69.2% | 1.01 (0.39; 2.42)/50.4% | 1.15 (0.63; 2.13)/67.6% | 0.81 (0.37; 1.74)/70.3% |
| | Severe vs No symptoms | | | | | 1.24 (0.46; 3.35)/66.3% | 2.38 (0.67; 7.73)/91.3% | 2.07 (0.78; 5.46)/93.0% | 0.34 (0.07; 1.34)/93.5% |
| | Died vs No symptoms | | | | | 0.55 (0.13; 2.08)/80.7% | 0.62 (0.11; 3.06)/71.5% | 1.49 (0.42; 5.14)/73.3% | 1.74 (0.44; 6,37)/79.3% |
| | Don't know vs No symptoms | | | | | 0.82 (0.45; 1.52)/73.3% | 0.88 (0.35; 2.23)/59.9% | 0.92 (0.49; 1.74)/60.1% | 1.48 (0.71; 3.14)/85.4% |
