# Supplementary material for: Effects of COVID-19 contagion in cohabitants and family members on mental health and academic self-efficacy among university students in Sweden: a prospective longitudinal study
Source: BMJ Open. 2024 Mar 12;14(3):e077396. doi: 10.1136/bmjopen-2023-077396 (PMC10936505; doi:10.1136/bmjopen-2023-077396)
Supplement: Supplementary data [file bmjopen-2023-077396supp011.pdf]

BMJ Publishing Group Limited (BMJ) disclaims all liability and responsibility arising from any reliance placed on this supplemental material which has been supplied by the author(s)


**Supplementary Table 4.** Contagion in family members at baseline and at 5-months follow-up in relation to self-reported change in mental health at 5-months and 10-months follow-ups. Reported are medians of the marginal posterior distributions of odds ratios with 2.5% and 97.5% percentiles, followed by posterior probability that the odds ratio is greater or less than 1 (in direction of the median).

| | | Self-reported change in mental health | | | | | |
|---|---|---|---|---|---|---|---|
| | | 5-months follow-up | | | 10-months follow-up | | |
| | | Worse vs. No change | Better vs. No change | Both vs. No change | Worse vs. No change | Better vs. No change | Both vs. No Change |
| Self-reported symptoms of Covid-19 contagion in a family member at baseline | Mild vs No symptoms | 1.38 (0.80; 2.37)/87.6% | 0.95 (0.41; 2.15)/54.4% | 1.37 (0.76; 2.46)/85.1% | 1.27 (0.67; 2.42)/76.3% | 0.55 (0.17; 1.60)/85.8% | 1.38 (0.70; 2.76); 81.7% |
| | Moderate vs No symptoms | 1.18 (0.63; 2.18)/69.9% | 1.62 (0.69; 3.70)/86.7% | 0.69 (0.33; 1.36)/85.7% | 0.69 (0.35; 1.35)/85.9% | 1.16 (0.41; 3.05)/61.5% | 0.73 (0.35; 1.54); 79.7% |
| | Severe vs No symptoms | 2.15 (0.65; 7.17)/89.7% | 0.54 (0.09; 2.76)/76.9% | 0.96 (0.25; 3.57)/52.6% | 2.84 (0.61; 14.30)/90.8% | 0.44 (0.04; 4.20)/75.7% | 1.60 (0.28; 8.88); 70.1% |
| | Died vs No symptoms | 1.43 (0.34; 6.05)/68.8% | 0.93 (0.17; 4.75)/53.3% | 1.39 (0.33; 6.03)/67.0% | 1.30 (0.21; 8.15)/61.0% | 1.45 (0.15; 12.0)/63.0% | 2.64 (0.43; 17.28)/85.1% |
| | Don’t know vs No symptoms | 0.70 (0.38; 1.27)/88.3% | 0.69 (0.27; 1.70)/78.5% | 0.85 (0.45; 1.60)/68.7% | 0.60 (0.31; 1.15)/94.0% | 0.96 (0.33; 2.65)/53.1% | 0.76 (0.38; 1.52)/77.3% |
| Self-reported symptoms of Covid-19 contagion in a family member at five months post-baseline | Mild vs No symptoms | | | | 0.89 (0.54; 1.47)/67.9% | 0.62 (0.24; 1.47)/85.8% | 1.18 (0.69; 2.03)/73.1% |
| | Moderate vs No symptoms | | | | 0.89 (0.51; 1.56)/66.1% | 0.51 (0.17; 1.34)/90.9% | 0.85 (0.46; 1.58)/69.3% |
| | Severe vs No symptoms | | | | 1.68 (0.66; 4.42)/85.7% | 1.23 /0.29; 4.48)/61.3% | 1.46 (0.53; 4.05)/77.2% |
| | Died vs No symptoms | | | | 0.70 (0.20; 2.33)/71.9% | 1.90 (80.4; 7.58)/80.4% | 0.72 (0.19; 2.53)/60.4% |
| | Don’t know vs No symptoms | | | | 0.81 (0.46; 1.42)/77.3% | 0.62 (0.23; 1.63)/83.1% | 0.69 (0.37; 1.32)/87.0% |
